# Assessment of soft error risks to cardiac implantable electronic devices for boron neutron capture therapy using field-programmable gate arrays

**DOI:** 10.1007/s11604-026-01993-9

**Published:** 2026-04-25

**Authors:** Takayuki Yagihashi, Makoto Sakai, Masashi Yamanaka, Yudai Kawakami, Reika Imazu, Kazunori Nitta, Takahiro Shimo, Shintaro Shiba

**Affiliations:** 1 Department of Medical Physics, Shonan Kamakura General Hospital, Kamakura, Kanagawa Japan; 2 Department of Radiological Sciences, Graduate School of Human Health Sciences, Tokyo Metropolitan University, Tokyo, Japan; 3 Radiological Research Division, Shonan Research Institute of Innovative Medicine, Shonan Kamakura General Hospital, Kamakura, Kanagawa Japan; 4 Department of Health Sciences, Faculty of Medical Sciences, Kyushu University, Fukuoka, Japan; 5 Department of Radiation Oncology, Gunma University Graduate School of Medicine, Gunma, Japan; 6 Department of Radiation Oncology, Shonan Kamakura General Hospital, Kamakura, Kanagawa Japan

**Keywords:** Boron neutron capture therapy, Cardiac implantable electronic device, Field-programmable gate array, Neutron irradiation, Soft error, Radiotherapy safety

## Abstract

**Purpose:**

Boron neutron capture therapy (BNCT) is a promising cancer treatment; however, it poses the risk of soft errors in cardiac implantable electronic devices (CIEDs). This study aimed to assess the occurrence of soft errors in CIEDs during BNCT and to correlate these errors with the neutron flux.

**Materials and methods:**

A field-programmable gate array (FPGA) was used as a surrogate for CIED to measure the soft errors under thermal and epithermal neutron irradiation. The neutron flux at the FPGA position was assessed using gold wire activation, and the effective soft-error cross-section was calculated.

**Results:**

The neutron flux exhibited a clear inverse relationship with distance from the irradiation center. The number of soft errors observed in the FPGA mirrored this trend, showing a significant reduction as the distance from the beam center increased. A strong linear correlation was identified between the thermal neutron flux and soft-error rate, and a consistent reaction cross-section was derived.

**Conclusion:**

This study provides foundational data on soft-error risks in electronic devices during BNCT. Our findings indicate that increasing the distance from the beam center significantly reduces the soft-error rate. These insights are crucial for developing robust radiotherapy safety guidelines for patients with implanted electronic devices.

**Supplementary Information:**

The online version contains supplementary material available at 10.1007/s11604-026-01993-9.

## Introduction

Boron neutron capture therapy (BNCT) is a targeted cancer treatment that utilizes the reaction between boron-10 and neutrons to generate high linear energy transfer α-particles and lithium nuclei. The short penetration ranges of these particles (~ 9–10 μm for α-particles and 4–5 μm for lithium nuclei) are used to destroy cancer cells selectively while minimizing damage to surrounding healthy tissues [1]. BNCT has shown efficacy in the treatment of brain [2, 3], head, and neck cancers [4, 5]. The introduction of epithermal neutrons together with accelerator-based BNCT (AB-BNCT) has broadened the clinical applications of the therapy by enabling hospital-based implementation and improving accessibility [6–12].

However, exposure to neutrons can induce transient malfunctions (or soft errors) in semiconductor devices [13–15]. Unlike hard errors, soft errors do not cause permanent damage, but may lead to critical failures. Reports of cardiac implantable electronic device (CIED) malfunctions, including life-threatening events such as ventricular tachycardia requiring cardiopulmonary resuscitation, have raised significant concerns [16, 17]. Previous studies have emphasized the role of neutrons, particularly thermal neutrons, in increasing the probability of soft errors [18–21]. For instance, according to Koivunoro et al., commercial pacemakers experience severe malfunction following irradiation and significant residual activation, demonstrating the risks to CIEDs in the irradiation field [22].

As applications of BNCT expand to diverse tumor sites [22–25], patients with CIEDs can also undergo this therapy, requiring their devices to be positioned outside the primary irradiation field. Nevertheless, studies including systematic evaluations of soft-error risks under such conditions remain scarce. Addressing this gap is essential for ensuring safe treatment in a growing patient population that relies on CIEDs [26, 27].

Therefore, this study aimed to assess the occurrence of soft errors during BNCT by focusing on in-field and out-of-field exposure. These errors were then correlated with the neutron flux. We used field-programmable gate arrays (FPGAs) as surrogates for CIEDs. Overall, this study provides new insights into the risks of BNCT-induced soft errors and supports the development of evidence-based strategies to expand the clinical use of BNCT safely.

## Methods

### Radiation source and beam characteristics

#### AB-BNCT system

An AB-BNCT system (nuBeam, Neutron Therapeutics LLC., Danvers, MA, USA) was used as the neutron source. In this system, protons were accelerated using an electrostatic accelerator and directed towards a lithium target to produce neutrons. Subsequently, the generated neutrons were moderated by magnesium fluoride (MgF_2_) and shaped by a collimator prior to irradiation.

#### Irradiation conditions

The proton beam was accelerated to an energy of 2.6 MeV, which was optimized for efficient neutron production using a lithium target. In the experiment, the average current was set to 30 mA.

The neutron beam collimation was manually adjusted using collimators with different diameters (11, 14, and 20 cm).

### Neutron flux measurements

The neutron flux at the FPGA position was assessed using gold wire activation. A thin gold wire (diameter: 0.25 mm, length: 10 cm, purity: 99.95%; Nilaco Corporation, Tokyo, Japan) was cut into 10 mm segments and placed at the same location as the FPGA within the polymethyl methacrylate (PMMA) block. Measurements were performed with and without a cadmium cover (thickness: 0.5 mm) (Nilaco Corporation) to differentiate between the thermal and epithermal neutron components.

The cadmium ratio was determined by comparing the induced activity in the bare and cadmium-covered gold wires, enabling an estimation of the thermal neutron contribution. The gold reaction rate (RR) was calculated as follows:

1 $$\:\begin{array}{*{20}c} {RR = \frac{{\lambda \:C}}{{\epsilon\:\gamma \:N_{0} \left( {e^{{ - \lambda \:t_{1} }} - e^{{ - \lambda \:t_{2} }} } \right)\sum \: _{n}^{{i = 1}} \left[ {\frac{{Q_{i} }}{{\Delta \:t}} \cdot \:\left( {e^{{ - \lambda \:\left( {n - i} \right) \cdot \:\Delta \:t}} - e^{{ - \lambda \:\left( {n - i + 1} \right) \cdot \:\Delta \:t}} } \right)} \right]}},} \\ \end{array}$$

where *λ*, *ε*, *γ*, *N*_0_, and *C* are the decay constant, detection efficiency, γ-ray emission ratio, total number of atoms in gold, and total photon-peak counts, respectively. In addition, *n* and *i* represent the numbers of trend data points during irradiation and data points, respectively. As the RR is calculated as the value for operation at 30 mA in the accelerator, *Q*_*i*_ is a correction value for the variation of the current, *n·Δt* is the irradiation time, and *t*_1_ and *t*_2_ are the start and end times of the measurement from the end of irradiation, respectively. The irradiation charge was 54 C. Measurement uncertainties were estimated based on the methods described in our previous study, which involved evaluations of the counting, reproducibility, and standard source uncertainties [28].

### FPGA utilization in soft-error measurements

#### FPGAs

FPGAs are integrated circuits that are reconfigurable following manufacturing [20]. These devices are widely used in various electronic applications, including CIEDs. FPGAs contain multiple memory units that are susceptible to radiation-induced errors owing to their architecture. Moreover, FPGAs have been employed in previous studies to evaluate soft errors induced by cosmic rays in electronic components [20, 29]. Therefore, we used an FPGA as a surrogate for CIEDs to quantify the number of soft errors generated under neutron irradiation.

#### Soft-error measurements

The soft errors generated via neutron irradiation were measured using an FPGA (Cmod S7, Digilent) surrogate. First, 9.93 Mb of configuration memory bits were written onto the FPGA using Vivado 2019.2 (Xilinx). Subsequently, the FPGA was placed in a phantom and irradiated with neutrons for 3–10 min, considering the collimator diameter and distance from the isocenter (as detailed in Supplementary Table 1).

Soft-error measurements were performed following established methodologies reported in previous radiotherapy and CIED-related studies [30, 31]. The configuration memory was initialized with a predominantly zero-valued bit pattern. Owing to implementation constraints of the programming procedure, only a small fraction of bits was set to “1.” The FPGA was then powered and operated during irradiation. User logic was clocked and active (LED blinking). Configuration scrubbing and error-correction mechanisms were not enabled during the irradiation experiments. Therefore, the configuration memory remained static throughout the irradiation, which allowed radiation-induced bit flips to be directly detected during the post-irradiation readback. As the configuration memory itself was not modified,, the potential pattern dependency of soft errors was not evaluated.

Following irradiation, the configuration memory was read back and compared with the pre-irradiation reference to identify persistent bit changes. These persistent configuration memory bit flips were counted as soft errors. The evaluated memory corresponded exclusively to SRAM-based configuration bits and did not include other memory resources such as block RAM or flip-flops.

#### Experimental setup

**Fig. 1:**
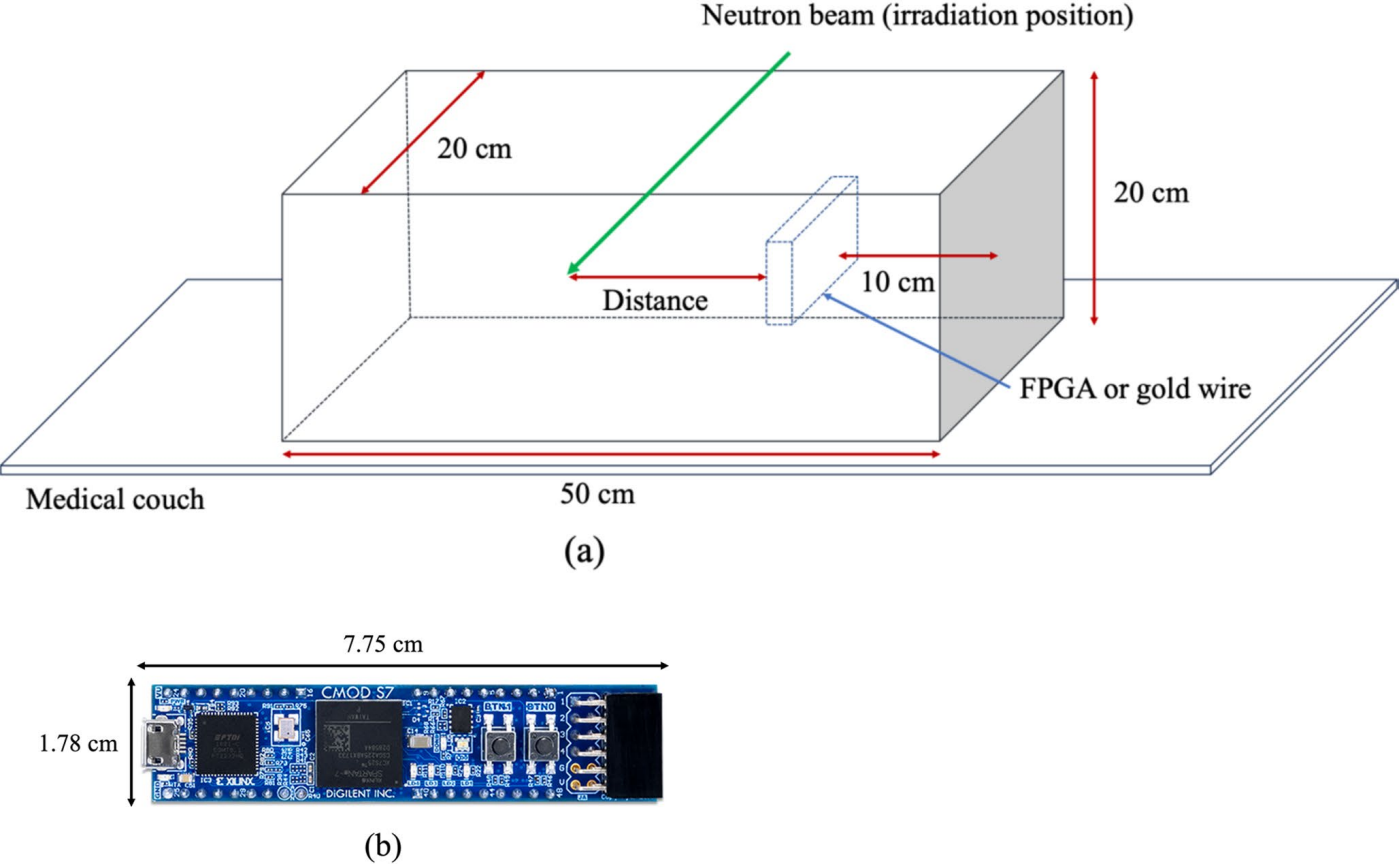
**a** Experimental setup and **b** FPGA board used

FPGAs were embedded at the center of a PMMA block with dimensions of 20 × 20 × 50 cm^3^ (Fig. 1) to measure soft errors under neutron irradiation. The distance from the FPGA to the collimator center (Fig. 1a) was systematically varied from 0 to 5, 10, 15, 20, and 30 cm. The number of memory bit flips (soft errors) was recorded for each condition. Each measurement was repeated thrice to ensure reliability.

### Soft-error cross-section calculations

For each irradiation condition, the observed number of soft errors was normalized by the irradiation time and total number of configuration memory bits to obtain a soft-error rate per bit. This rate was used to derive the effective soft-error cross-section. Because the rate is defined per unit time, its linear dependence on thermal neutron flux is equivalent to a fluence-based formulation. The effective soft-error cross-section was derived from the slope of a weighted linear regression of the soft-error rate per bit as a function of the thermal neutron flux, constrained to pass through the origin. Statistical uncertainties were estimated assuming Poisson-distributed soft-error counts.

## Results

### Thermal neutron flux measurements

**Fig. 2:**
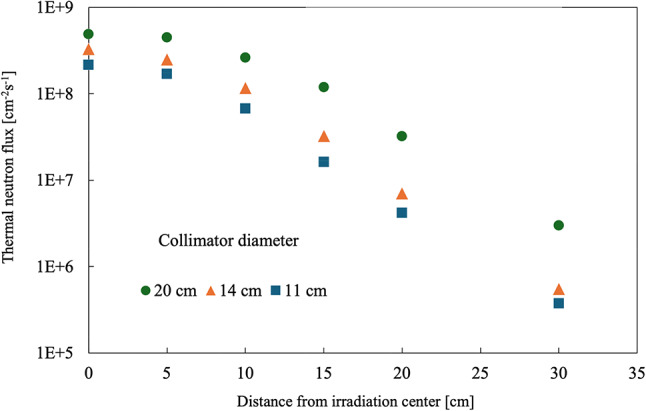
Neutron flux measurements as a function of the distance from the irradiation center for various collimator diameters

The thermal neutron flux measured at different distances from the beam center for collimator sizes of 11, 14, and 20 cm is shown in Fig. 2. The flux decreased with an increase in the distance from the irradiation center. The highest flux was observed at 0 cm, with values of 2.16 × 10^8^, 3.30 × 10^8^, and 4.85 × 10^8^ cm^−2^s^− 1^ for collimator diameters of 11, 14, and 20 cm, respectively. The detailed results are summarized in Table 2 of Online Resource 1.

**Fig. 3:**
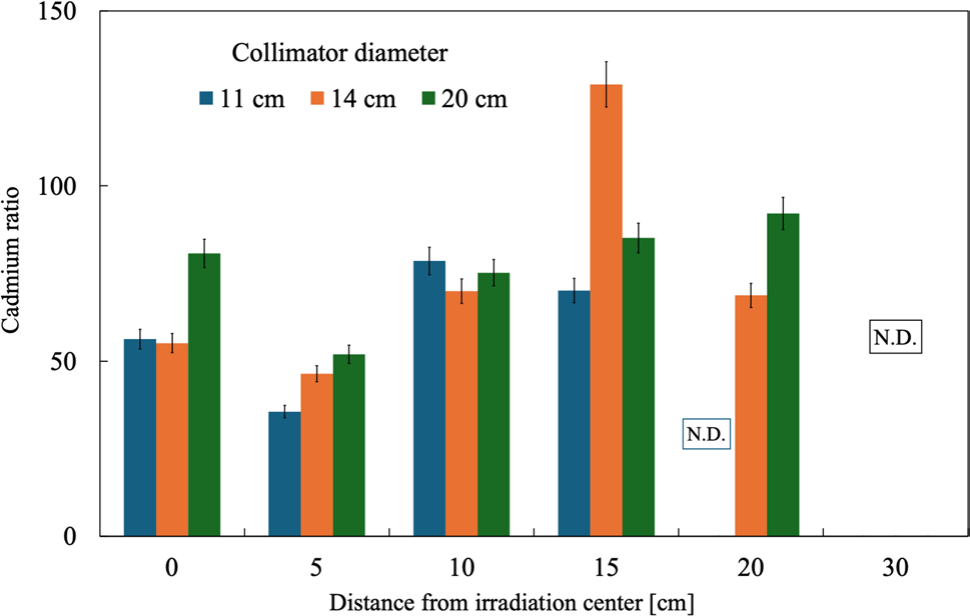
Cadmium ratio as a function of the distance from the irradiation center for three different collimator diameters. “N.D.” implies that the cadmium ratio was not detected at the corresponding position

The cadmium ratio obtained from the gold activation measurements at different distances from the beam center for different collimator sizes is shown in Fig. 3. For example, for the 11 cm collimator at a 20 cm distance, no gamma peaks attributable to ^198^Au activation were observed in the HPGe spectra for the cadmium-covered gold wire; therefore, the cadmium ratio was classified as not detected (N.D.). This result indicates that the induced activity was below the detection limit under the present counting conditions, suggesting a negligible contribution of epithermal neutrons at this location.

### Soft-error measurements

**Fig. 4:**
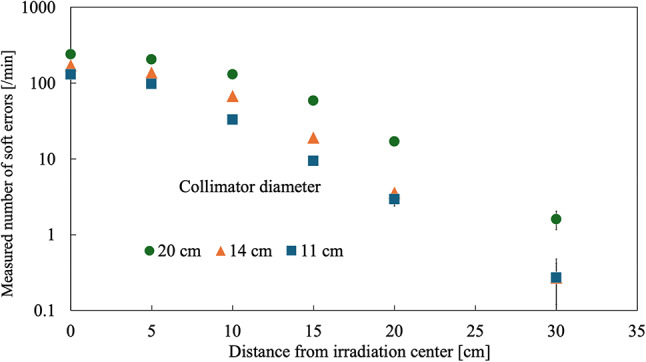
Soft-error measurements as a function of the distance from the irradiation center for various collimator diameters

The number of soft errors observed in the FPGA under various irradiation conditions is shown in Fig. 4. The number of soft errors, which was correlated with the distance from the beam center, decreased as the distance increased. At 0 cm, the soft-error counts were 129.00, 170.89, and 238.22 for collimator diameters of 11, 14, and 20 cm, respectively. A correlation with the collimator size was also observed. The observed decrease in soft error counts followed the expected trend based on thermal neutron flux reduction. The detailed results are summarized in Table 3 of Online Resource 1.

### Soft-error cross-section analysis

**Fig. 5:**
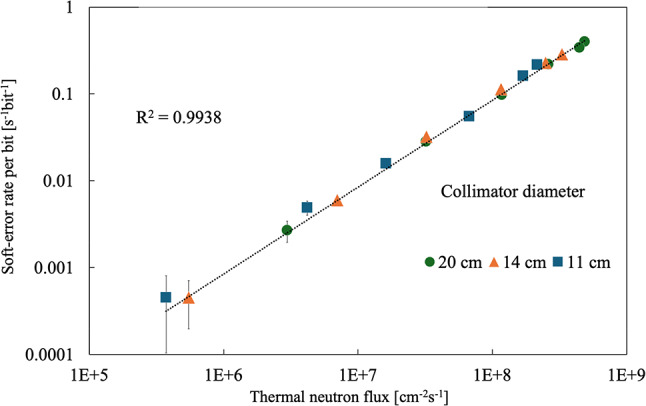
Measured soft-error rate per bit as a function of the thermal neutron flux. The data were obtained for various collimator diameters

The relationship between the thermal neutron flux and soft-error rate per bit was evaluated to quantify the severity of the neutron-induced soft-error generation. A strong linear correlation was observed between the two quantities (Fig. 5) across all experimental conditions (R² = 0.9938).

Using a weighted linear regression of the soft-error rate per bit as a function of thermal neutron flux, with the intercept constrained to zero, the resulting soft-error cross section was 9.17 × 10⁻¹⁰ cm²/Mb, with a corresponding 95% confidence interval of 8.72–9.62 × 10⁻¹⁰ cm²/Mb.

## Discussion

In this study, a strong quantifiable relationship was observed between thermal neutron flux and soft-error occurrence in FPGAs. The thermal neutron flux and soft-error counts both decreased exponentially with the distance from the irradiation center across all collimator sizes. This consistency suggests that thermal neutrons are the primary source of soft errors, which is consistent with the findings of previous studies [32, 33].

Notably, the accelerator-based BNCT beam used in this study is designed to provide a high epithermal neutron flux with minimized fast neutron and gamma-ray dose components, as reported in similar clinical AB-BNCT studies [34]. Although components from fast neutrons and gamma rays cannot be strictly excluded, their influence is expected to be negligible compared with that of thermal neutrons under the irradiation conditions of this study.

The log–log analysis (Fig. 5) provided clear evidence of a proportional relationship, with a near-perfect linear correlation (R² = 0.9938) between the thermal neutron flux and soft-error rate per bit across all experimental conditions, regardless of the collimator sizes and distances. The soft-error cross-section was derived from a linear regression of the soft-error rate per bit as a function of the thermal neutron flux on linearly scaled data for quantitative evaluation. The resulting cross-section was of the order of 10⁻⁹ cm²/Mb and agreed with values reported for similar devices [29]. Importantly, the convergence of data from all three collimator sizes onto a single trend line highlights that soft error generation depends primarily on the total thermal neutron flux, rather than on specific beam configurations. This relationship remained consistent over approximately three orders of magnitude of thermal neutron flux, indicating stable neutron–device interaction behavior in the investigated range.

Importantly, the reported cross-section represents an effective value corresponding to the specific logic-state distribution of the configuration memory used in this study, in which most bits were initialized to logic “0,” and a smaller fraction to logic “1.” As bit-flip probabilities are not necessarily symmetric with respect to the bit-flip direction, state-dependent sensitivity may influence the absolute value of the measured cross-Sect [35]. Nevertheless, this measurement provides a consistent and reproducible estimate of neutron-induced soft-error susceptibility at the semiconductor level under well-defined operating conditions.

From a clinical perspective, our findings highlight two important protective factors: distance and collimator size. The soft error count was reduced by ~ 99% at 30 cm compared with that at the beam center and decreased by an additional order of magnitude for every 10 cm increase in distance. In this study, this distance was defined as the separation from the beam central axis. CIEDs are most commonly implanted in the left infraclavicular region. Clinically, the separation between CIEDs and head and neck treatment targets is often limited; for example, the reported distances between the device and clinical target volume are typically a few centimeters (approximately 3–10 cm) for head and neck cases [36]. In brain cancer cases, the distance from the treatment area center to the thyroid gland (defined with respect to the beam central axis) has been used as a conservative estimate of the clinically relevant separation. In a clinical study of intracranial CyberKnife treatments, this distance was reported to range from 14 to 22 cm [37]. This separation is substantially larger than that typically encountered in head and neck radiotherapy. Because neutron fluence decreases rapidly with an increasing distance from the irradiation field, treatment planning strategies that place the target closer to the collimator periphery can further increase the effective separation between the beam center and CIED. Accordingly, the distances investigated in this study extend beyond those commonly encountered in head and neck and brain radiotherapy, and the reported ~ 99% reduction in the soft-error count at 30 cm should be interpreted as a relative decrease under specific irradiation conditions, rather than an absolute guarantee of device safety.

The distance-based trends should be interpreted as facility normalized as opposed to universally applicable absolute values. Although accelerator-based BNCT systems employ different source neutron spectra and beam-shaping assemblies, recent multi-facility studies have demonstrated that the constrained optimization of beam shaping assemblies leads to highly similar in-phantom thermal neutron fluence depth profiles across contemporary AB-BNCT systems. This physical similarity supports the transferability of the observed qualitative relationships between thermal neutron flux and soft-error occurrence, while acknowledging that quantitative risk thresholds remain facility dependent [38].

Our results demonstrated that even modest increases in the clinically relevant distance can markedly reduce risks to implanted electronic devices. In addition, the 20 cm collimator produced more soft errors than the 14 and 11 cm collimators at equivalent distances, indicating a consistent increase in risk with collimator sizes. The findings suggest that risk requires estimation based on the prescribed dose and tumor position, without detailed thermal neutron flux calculations, thereby providing a practical framework for clinical assessment. The study results provide quantitative evidence to support practical field- and distance-based guidelines for clinical BNCT planning.

The results indicate that minimizing the field size and maximizing the distance between the CIED and irradiation field are effective strategies for risk reduction. As pacemakers are typically located near the left clavicle, their exposure to irradiation depends strongly on patient positioning. For example, when a patient is treated in the supine position with a horizontal beam directed to the head and neck, the CIEDs may lie in the irradiation path. Switching to a sitting position can move the device outside the treatment field, effectively increasing the distance and thereby reducing the risk [39]. Therefore, although BNCT is often contraindicated for patients with CIEDs owing to the high neutron flux [15, 40], careful risk management using distance- and field-based strategies could mitigate soft-error risks and potentially expand their applications. Compared with other radiotherapy modalities, such as carbon ion radiotherapy and high-energy X-ray therapy, BNCT poses a higher risk of soft errors owing to direct neutron irradiation, rather than lower-flux secondary neutrons [31, 41]. In our results, soft-error occurrence was strongly correlated with the thermal neutron component within the sensitivities of the study measurements, which differs from findings in particle beam therapy in which higher-energy neutrons are known to dominate soft error mechanisms [30]. These findings underscore the need for BNCT-specific shielding approaches. For example, attaching thin films containing gold, lithium, or boron to the device case could significantly reduce the risk, while also protecting against naturally occurring soft errors from cosmic radiation [42–44].

This work has several limitations. The results were obtained from only one type of FPGA, and the soft-error probability was not evaluated using a clinical device. FPGA manufacturers have reported failures over time [45]. The FPGA-based surrogate approach uses measurements that target persistent configuration memory changes and does not capture other clinically relevant CIED behaviors, such as transient functional disturbances, device resets, sensing or pacing errors, or permanent hardware failures. In addition, the evaluated memory corresponded only to configuration SRAM bits, and other memory structures and dynamic circuit activity were not investigated. Furthermore, the configuration memory was initialized with a fixed data pattern, and potential state-dependent flip sensitivity (e.g., asymmetry between 0→1 and 1→0 transitions), as commonly reported in FPGA-based soft-error studies using neutron irradiation [35], was not evaluated. Therefore, although the FPGA provides a sensitive and reproducible surrogate for neutron-induced soft errors at the semiconductor level, extrapolation to specific CIED architectures requires additional consideration.

The risk of soft errors varies with the semiconductor process size and circuit design. The risk during clinical exposure may also vary depending on the CIED process size, which differs among products; moreover, semiconductors of various process sizes can be used in a single product. The risk of soft errors requires assessments via simulations or simple measurements of risk coefficients similar to those of CIEDs.

In addition, our experiments were conducted in controlled laboratory conditions, which may not exactly replicate the varied radiation environments that are encountered in real-world scenarios. Clinical applications may involve different temporal exposure patterns compared to repetitive experimental irradiation, potentially affecting cumulative soft error rates. The linear relationship without saturation in our experiments indicates that the study equipment operated in a regime of countable soft errors, demonstrating the robustness of our method. In the future, we will verify that the same measurements can be performed under significantly high-flux conditions that are comparable to the highest flux levels used in BNCT.

## Conclusions

In this study, the relationship between the thermal neutron flux and soft-error occurrence in FPGAs during BNCT was studied. Consequently, a linear correlation with a cross-section of 9.17 × 10^− 10^ cm^2^/Mb was obtained. The results revealed a 99% reduction in the soft-error count at a distance of 30 cm from the beam center, underscoring the importance of maintaining a safe distance from the irradiation field and an appropriate collimator size to minimize soft errors in CIEDs during BNCT. The findings of this study provide essential insights for the development of safety guidelines for radiotherapy.

## Supplementary Information

Below is the link to the electronic supplementary material.

Supplementary Material 1

Supplementary Material 2

Supplementary Material 3

## Data Availability

Data supporting the findings of this study are available from the corresponding author upon reasonable request.
