# Supplementary material for: Assessment of soft error risks to cardiac implantable electronic devices for boron neutron capture therapy using field-programmable gate arrays
Source: Jpn J Radiol. 2026 Apr 25;44(8):1477–84. doi: 10.1007/s11604-026-01993-9 (PMC13400480; doi:10.1007/s11604-026-01993-9)
Supplement: Supplementary file 1 — Supplementary Material 1 [file 11604_2026_1993_MOESM1_ESM.docx]

Supplementary Table 1. Neutron irradiation times (in minutes) for the FPGA for various collimator diameters and distances from the isocenter.
