# Supplementary material for: Assessment of soft error risks to cardiac implantable electronic devices for boron neutron capture therapy using field-programmable gate arrays
Source: Jpn J Radiol. 2026 Apr 25;44(8):1477–84. doi: 10.1007/s11604-026-01993-9 (PMC13400480; doi:10.1007/s11604-026-01993-9)
Supplement: Supplementary file 2 — Supplementary Material 2 [file 11604_2026_1993_MOESM2_ESM.docx]

Table S1. Measured neutron flux (cm^-2^s^-1^) as a function of distance and collimator size.
