# Supplementary material for: Assessment of soft error risks to cardiac implantable electronic devices for boron neutron capture therapy using field-programmable gate arrays
Source: Jpn J Radiol. 2026 Apr 25;44(8):1477–84. doi: 10.1007/s11604-026-01993-9 (PMC13400480; doi:10.1007/s11604-026-01993-9)
Supplement: Supplementary file 3 — Supplementary Material 3 [file 11604_2026_1993_MOESM3_ESM.docx]

Table S2. Measured soft error counts and standard deviation (SD) as a function of distance and collimator size.
